# Elevated plasma levels of Th17-related cytokines are associated with increased risk of atrial fibrillation

**DOI:** 10.1038/srep26543

**Published:** 2016-05-20

**Authors:** Na Wu, Bin Xu, Yuan Liu, Xinghua Chen, He Tang, Long Wu, Ying Xiang, Mengxuan Zhang, Maoqing Shu, Zhiyuan Song, Yafei Li, Li Zhong

**Affiliations:** 1 Department of Epidemiology, College of Preventive Medicine, Third Military Medical University, Chongqing 400038, People’s Republic of China; 2 Evidence-based Medicine and Clinical Epidemiology Center, Third Military Medical University, Chongqing 400038, People’s Republic of China; 3 Department of Cardiology, Southwest Hospital, Third Military Medical University, Chongqing 400038, People’s Republic of China; 4 Institute of Toxicology, College of Preventive Medicine, Third Military Medical University, Chongqing 400038, People’s Republic of China

## Abstract

We performed a matched case-control study using a propensity score matching, to assess the association of Th17-related cytokines, including interleukin (IL) 17A (IL-17A), IL-17F, IL-21, IL-22 and IL-6, along with interferon-γ (IFN-γ), IL-10, IL-9, and IL-4, with the risk of AF. A total of 336 patients with AF were matched 1:1 with patients without AF. Plasma levels of cytokines were measured using Luminex xMAP assays. The plasma levels of all examined cytokines were significantly higher in AF patients than controls (*P* < 0.05), and these cytokines were highly correlated with each other (*P* < 0.01). A multivariate conditional logistic regression analysis showed that elevated plasma levels of IL-17A, IL-17F, IL-21, IL-22, IFN-γ, IL-10, IL-9 and IL-6 were significantly associated with AF risk independently of potential confounders. There were no significant differences in plasma levels of examined cytokines between paroxysmal and chronic AF patients. IL-17A, IL-21, IL-10 and IL-6 levels were positively correlated with left atrial diameter; IL-17F level was negatively correlated with left ventricle ejection fraction among AF patients (*P* < 0.05). Elevated plasma levels of Th17-related cytokines were independently associated with increased an risk of AF; hence, Th17-related cytokines may be involved in the pathogenesis of AF.

Atrial fibrillation (AF) is the most common type of arrhythmia, accounting for approximately one-third of hospitalizations for cardiac rhythm disturbances. AF is associated with an increased long-term risk of strokes, heart failures and all-cause mortality^1^. The pathogenesis of AF is multifactorial and has not been fully elucidated. Recently, several studies have been conducted regarding the role of inflammation in the development of AF^2^,^3^. Researchers have observed inflammatory infiltrates in atrial biopsies from AF patients and animal models. The circulating levels of some inflammatory cytokines including C-reactive protein (CRP) are associated with AF^2^,^3^. Moreover, several medications with established anti-inflammatory effects such as statins and corticosteroids have been reported to reduce the risk of AF^4^. Various immune cells may play critical roles in the pathogenesis of AF. However, to the best of our knowledge, which specific inflammatory cells and related cytokines contribute to the development of AF still remains unclear.

Naive T helper lymphocytes may differentiate into different lineages that are characterized by different secreted cytokines. CD4^+^ T helper 17 (Th17) cells are a subgroup of T cells that are characterized by the production of interleukin (IL)-17A and IL-17F^5^. Other effector cytokines secreted by Th17 include IL-21, IL-22, and IL-6^6^,^7^. Recent findings indicate the plasticity of Th17 cells^8^. In the absence of TGF-β but in the presence of IL-6, IL-1β, and IL-23, alternative Th17 cells produce higher levels of IFN-γ^9^. In contrast, in the presence of TGF-β, classical Th17 cells are able to produce IL-10 and IL-9^6^. While, in presence of IL-4, Th17 cells shift to the production of IL-4^10^. Th17 and its related cytokines have been reported to be pathogenic and autoreactive. They are associated with the pathogenesis of several cardiovascular diseases, including pulmonary arterial hypertension, atherosclerosis, hypertension, viral myocarditis, myocardial ischaemia/reperfusion injury, and dilated cardiomyopathy^11^,^12^,^13^. However, there is limited evidence suggesting a specific relationship between Th17 cells or Th17-related cytokines and AF. Investigating the entire family of Th17-related cytokines in AF, including characteristic effector cytokines such as IL-17A, IL-17F, IL-21 and IL-22, and other non-characteristic cytokines secreted by Th17, can provide a whole picture.

Therefore, we conducted a propensity-score matched case-control study to investigate the association of Th17-related cytokines, including IL-17A, IL-17F, IL-21, IL-22 and IL-6, along with IFN-γ, IL-10, IL-9, and IL-4, with the risk of AF. We further compared the differences of plasma levels of all examined cytokines among patients with different clinical types of AF, and examined the correlation of these cytokines with atrial remodeling.

## Results

The characteristics of the AF patients and the controls are presented in Table 1. After propensity score matching, the baseline characteristics were comparable between the groups. There were no significant differences in gender, hypertension, diabetes, smoking status, drinking status and BMI status between the groups. More individuals in AF group were taking statins and aspirin, and had coronary artery disease than control group. Moreover, the AF patients were significantly older than the controls (median age, 67 years vs 45 years; *P* < 0.001). When participants were stratified by age, there was no significant difference in age between the groups for those aged at least 60 years (Supplementary Table S1).

Levels of all examined cytokines, including IL-17A, IL-17F, IL-21, IL-22, IFN-γ, IL-10, IL-9, IL-6 and IL-4, were significantly higher in the AF patients than in the controls (Table 1). Moreover, these cytokines had pair-wise correlations with Spearman’s rank correlation coefficients ranging from 0.625 to 0.966 (*P* < 0.01) (Supplementary Table S2).

The multivariate conditional logistic regression analysis showed that patients with elevated plasma levels of all examined cytokines except for IL-4 had a significantly higher risk of AF. After adjustment for age, use of statins, use of aspirin and history of coronary heart disease, the adjusted ORs for IL-17A, IL-17F, IL-21, IL-22, IFN-γ, IL-10, IL-9, and IL-6 were 2.58 (95% CI, 1.47–4.55; *P* = 0.001), 1.82 (95% CI, 1.05–3.16; *P* = 0.034), 2.08 (95% CI, 1.17–3.72; *P* = 0.013), 2.18 (95% CI, 1.26–3.79; *P* = 0.006), 2.47 (95% CI, 1.36–4.49; *P* = 0.003), 2.21 (95% CI, 1.25–3.94; *P* = 0.007), 2.74 (95% CI, 1.49–5.05; *P* = 0.001) and 2.50 (95% CI, 1.40–4.47; *P* = 0.002), respectively (highest vs lowest tertile) (Table 2). There were significant linear trends across categories for these eight cytokines. When participants were stratified by age, the results were not materially altered except for IL-17F, whose association became nonsignificant. (Supplementary Tables S3 and S4).

In order to further distinguish whether the association is due to the uneven distribution of age between two groups or not, we performed a sensitivity analysis. To make the age comparable between AF group and control group, we only select the controls who are at least 51 years old as a new control group. The mean age of the new control group is not significantly different from that of AF group. And all other characteristics except for hypertension history were comparable between AF patients and the new control group (Supplementary Table S5). The multivariate unconditional logistic regression analysis showed that significantly higher risk of AF was associated with elevated plasma levels of all examined cytokines. The results were not materially altered (Supplementary Table S6). To eliminate the potential confounding effect of hypertension, we stratified the participants by hypertension and only analyzed those with no history of hypertension. All of the characteristics were comparable between the groups and the results were still robust (data not shown).

We explored the differences in plasma levels of all examined cytokines between paroxysmal and chronic AF patients and found no significant differences in plasma levels of these cytokines (Table 3). To determine whether plasma levels of these cytokines correlated with atrial remodelling, we performed a partial Spearman’s correlation with adjustment for potential confounders to test the correlations between cytokine levels and echocardiographic parameters in the AF patients. Plasma levels of IL-17A, IL-21, IL-10 and IL-6 were significantly correlated with the left atrial diameter (LAD) (*r* = 0.118, 0.126, 0.117 and 0.134, respectively; *P* < 0.05) (Table 4). The level of IL-17F was positively correlated with the left ventricle diameter (LVD) and negatively correlated with the left ventricle ejection fraction (LVEF) (*r* = 0.128 and −0.130, respectively; *P* < 0.05). However, the correlation coefficients were relatively small (Table 4).

## Discussion

In this study, we determined elevated plasma levels of Th-17 related cytokines, including IL-17A, IL-17F, IL-21, IL-22 and IL-6, along with IFN-γ, IL-10 and IL-9, were significantly associated with increased AF risk independently of potential confounders. IL-17A, IL-21, IL-10 and IL-6 levels were positively correlated with LAD; the IL-17F level was negatively correlated with LVEF among AF patients. This is the first study that provides direct evidence on the association between the entire family of Th17-related cytokines and the risk of AF.

Recently, it has been proposed that inflammation has a possible pathogenic link to AF^14^, but it is still unknown which specific inflammatory cells or which related secreted cytokines contribute to the development of AF. Th17 cells have been recognized as a unique subset of CD4^+^ T cells that produce IL-17A, IL-17F, IL-21, IL-22 and IL-6^5^,^6^. Th17 cells and their effector cytokines play important roles in the pathogenesis of inflammation and autoimmunity as well as in host defence^7^.

In this study, we determined that elevated plasma levels of Th17-related cytokines including IL-17A, IL-17F, IL-21, IL-22 and IL-6, along with IFN-γ, IL-10 and IL-9, were independently associated with increased risk of AF. IL-17A and IL-17F are the characteristic Th17-derived effector cytokines, and are also the typical proinflammatory mediators. IL-17A plays a proatherogenic inflammatory role in pulmonary arterial hypertension, atherogenesis, myocardial ischaemia/reperfusion injury, dilated cardiomyopathy and heart failure^13^,^15^,^16^,^17^. Only one previous study has shown a relationship between IL-17A and AF, and that study has found that IL-17A is significantly increased in patients with AF^18^.

IL-21 can be produced by several populations of T cells, with Th17 cells producing the largest amount^19^. IL-21 together with TGF-β is able to induce Th17 differentiation^5^. IL-22 has been identified as a cytokine expressed by Th17 cells that has a synergistic effect with IL-17A or IL-17F. The expression levels of IL-21 and IL-22 are markedly increased in acute virus-induced myocarditis (AVMC), and IL-22 exacerbates the severity of AVMC^20^. Patients with acute coronary syndromes had significantly increased level of IL-22^21^.

Th17 cells also secrete IFN-γ, IL-10, IL-9, IL-6 and IL-4, although in a non-specific manner. Several studies have investigated the roles of IFN-γ and IL-10 in AF development and have demonstrated higher levels of IFN-γ and IL-10 in patients with AF or postoperative AF^22^,^23^. Our group has conducted a comprehensive meta-analysis to summarize the evidence regarding the association between a series of circulating inflammatory factors and AF occurrence, persistence and recurrence^24^. According to 52 studies, we have found that increased circulating IL-6 levels are significantly associated with AF risk. The results of these previous studies are consistent with our findings. With regard to IL-9, no other studies have reported an association between IL-9 and AF.

Higher levels of IL-4 were observed in AF patients than in controls, according to our results. However, after adjusting for age, history of coronary heart disease and use of some medications, no association was found between IL-4 and risk of AF, suggesting a high impact of confounders on this cytokine. Previous studies have reported similar results, and they have found no association between level of IL-4 and postoperative AF development or AF recurrence^22^,^25^. IL-4 is known to suppress the production of some inflammatory cytokines from monocytes and macrophages, and is shown to promote wound healing and tissue repair^26^. Therefore, it is possible that IL-4 does not contribute to AF, probably because of its role in anti-inflammatory responses.

The mechanism of these cytokines driving AF has not been elucidated yet. Some evidences suggest that inflammatory pathways are directly linked to ion channel dysfunction, connexin malfunction and matrix turnover, which lead to electrical and structural remodelling that predisposes an individual to AF^27^. Sodium and calcium ions are crucial to maintain the normal cardiac action potential, and some cytokines are known to provoke sodium and calcium channel dysfunction^28^,^29^. In addition, atrial conduction depends on the integrity of connexins. It has been reported that inflammatory processes are related to altered expression of connexins. Cytokines may disturb connexin integrity, which promotes AF^30^,^31^. Moreover, inflammation regulates extracellular homogeneity of atrial tissue and is firmly linked to fibrosis. Abnormal deposition of extracellular matrix (ECM) protein is the main characteristic of tissue fibrosis^32^. The MMPs and TIMPs are important enzymes regulating ECM metabolism and degrading collagen in the atria^33^. Some cytokines including IL-17A influence the expression of MMP in cardiac fibroblasts, and may contribute to myocardial fibrosis by influencing the MMP/TIMP system^34^,^35^.

Previous studies have demonstrated that patients with persistent or permanent AF have significantly higher level of CRP than paroxysmal AF patients, suggesting that the higher levels of inflammatory factors might be associated with the increased burden of AF^36^,^37^. However, in our study, we did not find any significant differences in examined cytokine levels between patients with paroxysmal and chronic AF; this finding is consistent with results from a previous study^18^. The precise mechanism needs to be further investigated.

Structural atrial remodelling provides a crucial substrate in the pathogenesis of AF. Previous studies have demonstrated that high levels of CRP and IL-18 are positively correlated with LAD, which may promote AF development^38^,^39^. In our study, we found that IL-17A, IL-21, IL-10 and IL-6 levels were positively correlated with LAD, and there was a negative correlation between the plasma level of IL-17F and LVEF among AF patients, suggesting that Th17-related cytokines may contribute to AF development through atrial remodelling. However the correlation coefficients were relatively small, indicating the impact of these cytokines may be small.

To our knowledge, this study is the first to examine the association between Th17-related cytokines and AF risk, using a propensity-score matched case-control study design and covering nine inflammatory factors. Given the current high incidence of AF, identifying the role of Th17-related cytokines in the development of AF has an important implication in understanding the aetiology and mechanisms underlying AF. However, several limitations of this study should be considered in interpreting our results. First, the mean age is not comparable between AF group and controls. To minimize the bias caused by the uneven distribution of age between the two groups, we used a multivariate conditional logistic regression to adjust for confounding factors including age. In addition, we conducted a stratified analysis and a sensitively analysis, and the results were not substantially altered, indicating the associations were robust. Second, selection bias might have been introduced because we recruited all of the cases and controls from a single tertiary medical center. Third, the results of our study can provide evidence of only an association between Th17-related cytokines and AF, instead of ascertaining a causal relationship. Whether the inflammatory effect is a consequence of AF, or whether the presence of a pre-existing systemic inflammatory status promotes AF development remains unclear. More studies are needed to judge whether this association is causal.

In the present study, we demonstrated that elevated plasma levels of Th17- related cytokines, including IL-17A, IL-17F, IL-21, IL-22 and IL-6, along with IFN-γ, IL-10 and IL-9, were independently associated with increased risk of AF, suggesting that Th17 cells may be involved in the pathogenesis of AF. These findings provide a basis for further understanding of the aetiology underlying AF. Future research is needed to elucidate the specific mechanism of Th17 and its related cytokines in the development of AF.

## Methods

### Study population

A 1:1 matched case-control study was conducted. Study participants were consecutively recruited from Southwest Hospital of the Third Military Medical University in Chongqing, China from December 2013 to January 2015. A total of 336 consecutive patients with AF admitted to the department of cardiology were enrolled. The diagnosis of AF subtypes was made according to the definition given by 2010 ESC guidelines^40^. AF that terminates spontaneously or with intervention within 7 days of onset was defined as paroxysmal AF. Patients with either persistent or permanent AF were defined as chronic AF, which indicates continuous AF that is sustained for more than 7 days, or a situation in which the patient and clinician make a joint decision to stop further attempts to restore and/or maintain sinus rhythm^40^.

Propensity-score matching was used to select controls to minimize selection bias due to perceived confounders. Potentially eligible controls were selected from 1257 individuals who had attended routine health check-up examinations during the same study period. All of them had no history of AF or any other arrhythmia. Individuals with intercurrent infective, inflammatory disorders and neoplastic disease were all excluded from case and control groups. AF patients were matched 1:1 to those without AF with the following variables as contributors to the propensity score: gender, a history of hypertension, a history of diabetes, smoking status, drinking status and body mass index (BMI). Overall 336 matched pairs were generated. All individuals provided written informed consent to participate. The study was approved by the Ethics Committee of the Third Military Medical University. The methods were carried out in accordance with the approved guidelines. There was no financial compensation.

### Participant information

Each subject underwent a structured interview to elicit a detailed information of demographic factors (age, gender, marriage status, education level and occupation), medical history (diagnosis years for arrhythmia, coronary heart disease, hypertension, diabetes and other diseases diagnosed by hospitals of second-tier or higher), medication use (medication use for arrhythmia, hypertension, diabetes, dyslipidemia, coronary heart disease and other diseases within the past one year), smoking status (smoking was defined as having smoked more than 100 cigarettes in the past), drinking status (drinking was defined as consuming alcohol at least once a week), physical activity and diet at enrolment. Structured questionnaires were administered in the same manner in cases and controls. Trained staff measured height and weight for all participants to calculate BMI (kg/m^2^), and participants were categorized as underweight (<18.5 kg/m^2^), normal (18.5 kg/m^2^ ≤ BMI < 25.0 kg/m^2^), overweight (25.0 kg/m^2^ ≤ BMI < 30.0 kg/m^2^), or obese (≥30.0 kg/m^2^). Echocardiography was performed during hospitalization for AF patients. Information regarding echocardiographic parameters was available for 319 AF patients. Echocardiographic parameters included in this study were left atrial diameter (LAD), right atrial diameter (RAD), left ventricle diameter (LVD) and left ventricle ejection fraction (LVEF).

### Measurement of cytokine levels

A fasting blood sample (5 mL) was drawn from each case (within 48 h of admission and before any treatment) and control (at the time of interview) and was centrifuged at 2000 g/minute for 15 minutes at 4 °C within 2 hours. Then, the plasma was aliquoted into 500-μL straws and frozen at −80 °C immediately after processing until use. Plasma levels of Th17-related cytokines, including IL-17A, IL-17F, IL-21, IL-22 and IL-6, along with IFN-γ, IL-10, IL-9 and IL-4 were measured by MILLIPLEX MAP Human Th17 Magnetic Bead Panel kits (Millipore, Billerica, MA., USA.) based on the Luminex xMAP technology (Luminex Corporation, Austin, TX., USA.). Plates were run on the Luminex MagPix machine (Luminex Corporation) and data were collected using Luminex xPONENT 4.2 software and were analysed using MILLIPLEX Analyst 5.1 software (Millipore). Concentrations of cytokines (pg/ml) were calculated using a standard curve. All samples were measured once. Two replicate quality control samples were run with each assay (replicate QC1 samples, low level; replicate QC2 samples, high level). The coefficients of variation (CVs) of replicate quality control samples were <10% for all cytokines.

### Statistical analysis

Propensity score matching was performed to adjust for potential confounders. A multivariable logistic regression was used to construct the propensity score for each participant using the following categorical covariates: gender, history of hypertension (yes or no), history of diabetes (yes or no), smoking status (smoker or not), drinking status (drinker or not) and BMI status (underweight, normal, overweight or obesity). We conducted propensity score matching with an SPSS R plug-in (SPSS R Essentials) using nearest neighbour matching and matching without replacement method, making the AF and control groups equal sizes by excluding non-matched cases. Overall 336 matched pairs were generated. The overall χ^2^ balance test was not significant [χ^2^(6) = 6.131, *P* = 0.409]. The L1 measure was larger in the unmatched sample (0.412) than in the matched sample (0.100), indicating that matching improved the overall balance. No covariate exhibited a large imbalance (standardized mean difference >0.25)^41^.

The descriptive statistics are presented as frequency counts and proportions for categorical data, and median and interquartile ranges (25th–75th percentile) for continuous variables that were not normally distributed. To test the differences in medians and proportions between AF cases and controls, we used a Wilcoxon signed rank test of paired samples and a chi-square test, respectively. A multivariate conditional logistic regression was used to evaluate the association between each cytokine individually and risk of AF to estimate adjusted odds ratios (OR) and 95% confidence intervals (CI), by adjusting for age, use of statins, use of aspirin and history of coronary heart disease. The cytokine levels were entered into the logistic regression model as ordinal categorical variables using tertiles with cutpoints based on the frequency distribution of both cases and controls, treating the lowest tertile as the reference group. Linear trends were calculated by treating the categorical variable as continuous in the logistic models. In order to distinguish whether the association is due to the uneven distribution of age between the two groups or not, we performed a stratified analysis and a sensitivity analysis. A multivariate unconditional logistic regression analysis was used to measure the association in stratified and sensitivity analysis.

To test the differences in cytokine levels between paroxysmal and chronic AF patients, a Mann-Whitney U test was used. For correlation studies of the cytokine levels with echocardiographic parameters in AF patients, we performed a partial Spearman’s correlation analysis with adjustment for age, gender, hypertension, diabetes, smoking status, drinking status, BMI, use of statins and use of aspirin. All statistical analyses were performed using SPSS statistical software (version 18.0; SPSS Inc., Chicago, IL, USA). A two-sided P value < 0.05 was considered to be statistically significant.

## Additional Information

**How to cite this article**: Wu, N. *et al.* Elevated plasma levels of Th17-related cytokines are associated with increased risk of atrial fibrillation. *Sci. Rep.* **6**, 26543; doi: 10.1038/srep26543 (2016).

## Supplementary Material

Supplementary Information

## Figures and Tables

**Table 1:** Characteristics of patients with AF and controls.

Characteristics*	AF cases (n = 336)	Controls (n = 336)	*P* value†
Age, y	67 (58–74)	45 (36–58)	<0.001
Gender, n (%)			0.073
Male	181 (53.9)	204 (60.7)	
Female	155 (46.1)	132 (39.3)	
Hypertension, n (%)			0.188
Yes	163 (48.5)	146 (43.5)	
No	173 (51.5)	190 (56.5)	
Diabetes, n (%)			0.740
Yes	49 (14.6)	46 (13.7)	
No	287 (85.4)	290 (86.3)	
Smoking status, n (%)			0.523
Smoker	121 (36.0)	129 (38.4)	
Non-smoker	215 (64.0)	207 (61.6)	
Drinking status, n (%)			0.806
Drinker	110 (32.7)	113 (33.6)	
Non-drinker	226 (67.3)	333 (66.4)	
BMI status, n (%)			0.886
BMI < 18.5	15 (4.5)	14 (4.2)	
18.5 ≤ BMI < 25.0	190 (56.5)	183 (54.5)	
25.0 ≤ BMI < 30.0	105 (31.3)	108 (32.1)	
BMI ≥ 30.0	26 (7.7)	31 (9.2)	
Taking statins, n (%)			<0.001
Yes	41 (12.2)	11 (3.3)	
No	295 (87.8)	325 (96.7)	
Taking aspirin, n (%)			<0.001
Yes	34 (10.1)	7 (2.1)	
No	302 (89.9)	329 (97.9)	
Coronary artery disease			<0.001
Yes	75 (22.3)	28 (8.3)	
No	261 (77.7)	308 (91.7)	
IL-17A, pg/mL	31.00 (15.98–46.73)	19.87 (12.82–33.43)	<0.001
IL-17F, pg/mL	0.01 (0.01–0.02)	0.01 (0.01–0.02)	0.001
IL-21, pg/mL	44.48 (25.33–67.13)	35.05 (20.39–50.66)	<0.001
IL-22, pg/mL	0.69 (0.32–0.95)	0.52 (0.14–0.80)	<0.001
IFN-γ, pg/mL	42.59 (21.86–67.13)	26.74 (14.84–48.51)	<0.001
IL-10, pg/mL	7.53 (2.08–15.73)	3.18 (0.94–8.95)	<0.001
IL-9, pg/mL	33.08 (11.89–56.25)	17.29 (6.81–35.68)	<0.001
IL-6, pg/mL	26.45 (9.98–47.47)	12.13 (4.07–28.85)	<0.001
IL-4, pg/mL	0.33 (0.11–0.54)	0.22 (0.09–0.40)	<0.001

AF, atrial fibrillation; IQR, interquartile range; BMI, body mass index; IL-17A, interleukin 17A; IL-17F, interleukin 17F; IL-21, interleukin 21; IL-22, interleukin 22; IFN-γ, interferon-γ; IL-10, interleukin 10; IL-9, interleukin 9; IL-6, interleukin 6; IL-4, interleukin 4.

^*^Entries are n (%) for categorical variables and median (5th percentile–75th percentile) for continuous variables as appropriate.

^†^Chi-square test for categorical variables, and Wilcoxon signed ranks test of paired samples for continuous variables.

**Table 2:** Associations between Th17-related cytokine levels with AF.

Cytokines*	Sample sizes, n		Conditional logistic regression	
	AF	Control	OR (95% CI)†	*P* value
IL-17A, pg/mL				
<16.30	87	138	Reference	
16.30–34.27	101	125	1.34 (0.76–2.37)	0.312
≥34.27	148	73	2.58 (1.47–4.55)	0.001
*P* value for trend				0.001
IL-17F, pg/mL				
<0.01	184	231	Reference	
0.01–0.02	68	50	1.36 (0.75–2.47)	0.320
≥0.02	82	53	1.82 (1.05–3.16)	0.034
*P* value for trend				0.028
IL-21, pg/mL				
<27.62	96	133	Reference	
27.62–50.66	106	120	1.20 (0.69–2.08)	0.518
≥50.66	133	82	2.08 (1.17–3.72)	0.013
*P* value for trend				0.015
IL-22, pg/mL				
<0.37	93	135	Reference	
0.37–0.80	107	118	1.30 (0.75–2.26)	0.352
≥0.80	134	81	2.18 (1.26–3.79)	0.006
*P* value for trend				0.006
IFN-γ, pg/mL				
<21.86	88	140	Reference	
21.86–49.57	103	117	1.24 (0.69–2.23)	0.474
≥49.57	144	78	2.47(1.36–4.49)	0.003
*P* value for trend				0.003
IL-10, pg/mL				
<2.24	87	139	Reference	
2.24–8.95	106	112	1.48 (0.84–2.62)	0.178
≥8.95	139	84	2.21 (1.25–3.94)	0.007
*P* value for trend				0.007
IL-9, pg/mL				
<12.41	89	137	Reference	
12.41–37.27	97	126	1.27 (0.74–2.19)	0.392
≥37.27	148	71	2.74 (1.49–5.05)	0.001
*P* value for trend				0.001
IL-6, pg/mL				
<9.40	83	145	Reference	
9.40–30.10	106	114	1.42 (0.80–2.52)	0.232
≥30.10	146	76	2.50 (1.40–4.47)	0.002
*P* value for trend				0.002
IL-4, pg/mL				
<0.16	106	134	Reference	
0.16–0.39	96	116	1.02 (0.59–1.75)	0.950
≥0.39	134	86	1.64 (0.95–2.83)	0.076
*P* value for trend				0.079

AF, atrial fibrillation; OR, odds ratio; CI, confidence interval; IL-17A, interleukin 17A; IL-17F, interleukin 17F; IL-21, interleukin 21; IL-22, interleukin 22; IFN-γ, interferon-γ; IL-10, interleukin 10; IL-9, interleukin 9; IL-6, interleukin 6; IL-4, interleukin 4.

^*^The cytokine levels were analysed as ordinal categorical variables using tertiles.

^†^A multivariable conditional logistic regression analysis was used to estimate the OR (95% CI) adjusted for age, history of coronary heart disease, use of statins and aspirin.

**Table 3:** Differences of Th17-related cytokine levels between paroxysmal AF and chronic AF patients.

Cytokines*	Paroxysmal AF (n = 84)	Chronic AF (n = 236)	*P* value†
IL-17A, pg/mL	28.75 (15.44–42.15)	31.00 (16.06–47.78)	0.330
IL-17F, pg/mL	0.01 (0.01–0.02)	0.01(0.01–0.03)	0.215
IL-21, pg/mL	43.07 (24.00–63.27)	44.48 (25.33–70.10)	0.426
IL-22, pg/mL	0.68 (0.22–0.91)	0.70 (0.35–0.97)	0.453
IFN-γ, pg/mL	42.59 (20.02–62.57)	42.59 (21.86–68.60)	0.288
IL-10, pg/mL	5.56 (1.28–14.42)	7.53 (2.36–15.73)	0.311
IL-9, pg/mL	27.47 (11.07–54.47)	33.44 (11.77–56.56)	0.398
IL-6, pg/mL	25.30 (8.10–42.15)	27.60 (10.20–48.50)	0.340
IL-4, pg/mL	0.30 (0.09–0.49)	0.34 (0.11–0.54)	0.298

AF, atrial fibrillation; IL-17A, interleukin 17A; IL-17F, interleukin 17F; IL-21, interleukin 21; IL-22, interleukin 22; IFN-γ, interferon-γ; IL-10, interleukin 10; IL-9, interleukin 9; IL-6, interleukin 6; IL-4, interleukin 4.

^*^The cytokine levels are expressed as median (25th percentile–75th percentile).

^†^Mann-Whitney U tests.

**Table 4:** Partial Spearman’s correlation between Th17-related cytokine levels and echocardiographic parameters in AF patients*.

	IL-17A	IL-17F	IL-21	IL-22	IFN-γ	IL-10	IL-9	IL-6	IL-4
LAD	**0.118**†	0.102	**0.126**†	0.049	0.096	**0.117**†	0.099	**0.134**†	0.110
	**(0.038)**	(0.073)	**(0.027)**	(0.394)	(0.091)	**(0.040)**	(0.083)	**(0.019)**	(0.054)
RAD	0.032	0.069	0.053	0.023	0.023	0.001	0.009	0.057	0.042
	(0.574)	(0.229)	(0.350)	(0.690)	(0.685)	(0.993)	(0.875)	(0.332)	(0.461)
LVD	0.077	**0.128**†	0.099	0.074	0.049	0.085	0.055	0.072	0.070
	(0.174)	**(0.025)**	(0.081)	(0.194)	(0.387)	(0.136)	(0.335)	(0.205)	(0.221)
LVEF	−0.034	**−0.130**†	−0.065	−0.062	−0.006	0.008	−0.017	−0.065	−0.031
	(0.552)	**(0.023)**	(0.257)	(0.280)	(0.912)	(0.887)	(0.767)	(0.259)	(0.589)

AF, atrial fibrillation; IL-17A, interleukin 17A; IL-17F, interleukin 17F; IL-21, interleukin 21; IL-22, interleukin 22; IFN-γ, interferon-γ; IL-10, interleukin 10; IL-9, interleukin 9; IL-6, interleukin 6; IL-4, interleukin 4; LAD, left atrial diameter; RAD, right atrial diameter; LVD, left ventricle diameter; LVEF, left ventricle ejection fraction.

^*^Partial Spearman’s correlation with adjustment for age, gender, hypertension, diabetes, smoking status, drinking status, BMI, use of statins and use of aspirin was used. Values are partial Spearman coefficients (P value).

^†^Correlation coefficients were significant at the 0.05 level (2-tailed).
