# Supplementary material for: Elevated plasma levels of Th17-related cytokines are associated with increased risk of atrial fibrillation
Source: Sci Rep. 2016 May 20;6:26543. doi: 10.1038/srep26543 (PMC4873818; doi:10.1038/srep26543)
Supplement: Supplementary Information [file srep26543-s1.doc]

**Elevated plasma levels of Th17-related cytokines are associated with increased risk of atrial fibrillation**

Na Wu, MD^1,2 *^; Bin Xu, MD^1,2 *^; Yuan Liu, MPH^1,2 *^; Xinghua Chen, MD^3^; He Tang, MD^4^; Long Wu, MPH^1,2^; Ying Xiang, MPH^1,2^; Mengxuan Zhang, MPH^1,2^; Maoqing Shu, MD^3^; Zhiyuan Song, MD, PhD^3^; Yafei Li, MD, PhD^1,2 #^; Li Zhong, MD, PhD^3 #^

*^1^ Department of Epidemiology, College of Preventive Medicine, Third Military Medical University, Chongqing 400038, People’s Republic of China*

*^2^ Evidence-based Medicine and Clinical Epidemiology Center, Third Military Medical University, Chongqing 400038, People’s Republic of China*

*^3^ Department of Cardiology, Southwest Hospital, Third Military Medical University, Chongqing 400038, People’s Republic of China*

*^4^ Institute of Toxicology, College of Preventive Medicine, Third Military Medical University, Chongqing 400038, People’s Republic of China*

^*^ These authors contributed equally to this work.

^#^ These authors jointly directed the project.

**Supplementary information**

**Supplementary Table S1.** Characteristics of patients with AF and controls stratified by age

**Supplementary Table S2.** Correlation coefficients among the Th17-related cytokines in all participants

**Supplementary Table S3.** Associations between Th17-related cytokine levels with AF in participants aged less than 60 years

**Supplementary Table S4.** Associations between Th17-related cytokine levels with AF in participants aged at least 60 years

**Supplementary Table S5.** Characteristics of all patients with AF and controls aged at least 51 years

**Supplementary Table S6.** Associations between Th17-related cytokine levels with AF in patients with AF and controls aged at least 51 years

Supplementary Table S1. Characteristics of patients with AF and controls stratified by age

| Characteristics * |  | Age < 60 years |  |  |  | Age > 60 years |  |
| --- | --- | --- | --- | --- | --- | --- | --- |
|  | AF cases  (n= 97) | Controls  (n= 254) | *P* value † |  | AF cases  (n= 239) | Controls  (n= 82) | *P* value † |
| Age, y | 51 (46-57) | 41 (34-48) | <0.001 |  | 71 (66-77) | 70 (64-75) | 0.137 |
| Gender, n (%) |  |  | 0.665 |  |  |  | 0.546 |
| Male | 61 (62.9) | 166 (65.4) |  |  | 120 (50.2) | 38 (46.3) |  |
| Female | 36 (37.1) | 88 (34.6) |  |  | 119 (49.8) | 44 (53.7) |  |
| Hypertension, n (%) |  |  | 0.165 |  |  |  | 0.007 |
| Yes | 25 (25.8) | 85 (33.5) |  |  | 138 (57.7) | 61 (74.4) |  |
| No | 72 (74.2) | 169 (66.5) |  |  | 101 (42.3) | 21 (25.6) |  |
| Diabetes, n (%) |  |  | 0.876 |  |  |  | 0.936 |
| Yes | 12 (12.4) | 33 (13.0) |  |  | 37 (15.5) | 13 (15.9) |  |
| No | 85 (87.6) | 221 (87.0) |  |  | 202 (84.5) | 69 (84.1) |  |
| Smoking status, n (%) |  |  | 0.913 |  |  |  | 0.125 |
| Smoker | 41 (42.3) | 109 (42.9) |  |  | 80 (33.5) | 20 (24.4) |  |
| Non-smoker | 56 (57.7) | 145 (57.1) |  |  | 159 (66.5) | 62 (75.6) |  |
| Drinking status, n (%) |  |  | 0.547 |  |  |  | 0.013 |
| Drinker | 42 (43.3) | 101 (39.8) |  |  | 68 (28.5) | 12 (14.6) |  |
| Non-drinker | 55 (56.7) | 153 (60.2) |  |  | 171 (71.5) | 70 (85.4) |  |
| BMI status, n (%) |  |  | 0.708 |  |  |  | 0.732 |
| BMI<18.5 | 3 (3.1) | 12 (4.7) |  |  | 12 (5.0) | 2 (2.4) |  |
| 18.5≤BMI<25.0 | 47 (48.5) | 135 (53.1) |  |  | 143 (59.8) | 48 (58.5) |  |
| 25.0≤BMI<30.0 | 36 (37.1) | 81 (31.9) |  |  | 69 (28.9) | 27 (32.9) |  |
| BMI≥30.0 | 11 (11.3) | 26 (10.2) |  |  | 15 (6.3) | 5 (6.1) |  |
| Taking statins, n (%) |  |  | 0.002 |  |  |  | 0.523 |
| Yes | 5 (5.2) | 1 (0.4) |  |  | 36 (15.1) | 10 (12.2) |  |
| No | 92 (94.8) | 253 (99.6) |  |  | 203 (84.9) | 72 (87.8) |  |
| Taking aspirin, n (%) |  |  | <0.001 |  |  |  | 0.373 |
| Yes | 5 (5.2) | 0 (0.0) |  |  | 29 (12.1) | 7 (8.5) |  |
| No | 92 (94.8) | 254 (100.0) |  |  | 210 (87.9) | 75 (91.5) |  |
| Coronary artery disease, n (%) |  |  | <0.001 |  |  |  | 0.286 |
| Yes | 11 (11.3) | 1 (0.4) |  |  | 64 (26.8) | 27 (32.9) |  |
| No | 86 (88.7) | 253 (99.6) |  |  | 175 (73.2) | 55 (67.1) |  |
| IL-17A, pg/mL | 31.00 (14.06-46.73) | 19.87 (12.14-32.60) | 0.001 |  | 31.00 (16.64-47.78) | 20.84 (14.53-38.76) | 0.006 |
| IL-17F, pg/mL | 0.01(0.01-0.03) | 0.01(0.01-0.02) | 0.007 |  | 0.01(0.01-0.02) | 0.01(0.01-0.02) | 0.034 |
| IL-21, pg/mL | 42.60 (22.52-66.41) | 35.05(20.39-51.21) | 0.012 |  | 44.97 (26.45-67.13) | 34.30(18.69-49.84) | 0.008 |
| IL-22, pg/mL | 0.69 (0.23-0.99) | 0.54 (0.14-0.80) | 0.007 |  | 0.70 (0.35-0.95) | 0.46 (0.12-0.83) | 0.013 |
| IFN-γ, pg/mL | 39.92 (17.28-67.15) | 26.74 (14.20-46.45) | 0.001 |  | 44.49 (22.85-67.50) | 29.17 (15.53-55.54) | 0.004 |
| IL-10, pg/mL | 6.90 (1.05-15.06) | 3.02 (0.88-8.67) | 0.003 |  | 7.86 (2.47-15.73) | 3.88 (1.36-10.42) | 0.011 |
| IL-9, pg/mL | 32.71 (7.73-55.05) | 17.29 (6.28-35.69) | <0.001 |  | 33.44 (12.15-56.87) | 18.64 (10.17-35.52) | 0.002 |
| IL-6, pg/mL | 25.30 (7.25-43.55) | 1.60 (3.55-27.60) | <0.001 |  | 27.60 (11.00-48.50) | 16.30 (5.35-33.08) | 0.002 |
| IL-4, pg/mL | 0.30 (0.09-0.54) | 0.22 (0.10-0.39) | 0.050 |  | 0.35 (0.12-0.54) | 0.21 (0.08-0.42) | 0.018 |

AF, atrial fibrillation; IQR, interquartile range; BMI, body mass index; IL-17A, interleukin 17A; IL-17F, interleukin 17F; IL-21, interleukin 21; IL-22, interleukin 22; IFN-γ, interferon-γ; IL-10, interleukin 10; IL-9, interleukin 9; IL-6, interleukin 6; IL-4, interleukin 4.

* Entries are n (%) for categorical variables and median (5th percentile-75th percentile) for continuous variables as appropriate.

† Chi-square test for categorical variables, and Mann-Whitney U test for continuous variables.

Supplementary Table S2. Correlation coefficients among the Th17-related cytokines in all participants*

|  | IL-17A | IL-17F | IL-21 | IL-22 | IFN-γ | IL-10 | IL-9 | IL-6 | IL-4 |
| --- | --- | --- | --- | --- | --- | --- | --- | --- | --- |
| IL-17A | 1.000 | 0.727 | 0.934 | 0.810 | 0.966 | 0.910 | 0.954 | 0.901 | 0.942 |
| IL-17F | - | 1.000 | 0.787 | 0.868 | 0.693 | 0.625 | 0.690 | 0.649 | 0.691 |
| IL-21 | - | - | 1.000 | 0.874 | 0.925 | 0.869 | 0.917 | 0.859 | 0.924 |
| IL-22 | - | - | - | 1.000 | 0.782 | 0.693 | 0.768 | 0.716 | 0.786 |
| IFN-γ | - | - | - | - | 1.000 | 0.905 | 0.945 | 0.899 | 0.933 |
| IL-10 | - | - | - | - | - | 1.000 | 0.926 | 0.853 | 0.887 |
| IL-9 | - | - | - | - | - | - | 1.000 | 0.889 | 0.940 |
| IL-6 | - | - | - | - | - | - | - | 1.000 | 0.885 |
| IL-4 | - | - | - | - | - | - | - | - | 1.000 |

IL-17A, interleukin 17A; IL-17F, interleukin 17F; IL-21, interleukin 21; IL-22, interleukin 22; IFN-γ, interferon-γ; IL-10, interleukin 10; IL-9, interleukin 9; IL-6, interleukin 6; IL-4, interleukin 4.

* A Spearman’s rank correlation analysis was performed. All correlation coefficients were significant at the 0.01 level (2-tailed).

Supplementary Table S3. Associations between Th17-related cytokine levels with AF in participants aged less than 60 years

| Cytokines ^*^ | Sample sizes, n | |  | Unconditional logistic regression† | |
| --- | --- | --- | --- | --- | --- |
|  | AF | Control |  | OR (95% CI) | *P* value |
| IL-17A , pg/mL |  |  |  |  |  |
| < 16.30 | 28 | 106 |  | Reference |  |
| 16.30-34.27 | 27 | 99 |  | 1.02 (0.50-2.07) | 0.954 |
| ≥34.27 | 42 | 49 |  | 2.77 (1.34-5.70) | 0.006 |
| *P* value for trend |  |  |  |  | 0.006 |
|  |  |  |  |  |  |
| IL-17F, pg/mL |  |  |  |  |  |
| < 0.01 | 49 | 170 |  | Reference |  |
| 0.01-0.02 | 19 | 43 |  | 1.14 (0.53-2.47) | 0.739 |
| ≥0.02 | 28 | 39 |  | 1.93 (0.96-3.89) | 0.064 |
| *P* value for trend |  |  |  |  | 0.075 |
|  |  |  |  |  |  |
| IL-21, pg/mL |  |  |  |  |  |
| < 27.62 | 29 | 101 |  | Reference |  |
| 27.62-50.66 | 28 | 89 |  | 1.00 (0.50-2.03) | 0.991 |
| ≥50.66 | 40 | 63 |  | 1.82 (0.90-3.67) | 0.094 |
| *P* value for trend |  |  |  |  | 0.097 |
|  |  |  |  |  |  |
| IL-22 , pg/mL |  |  |  |  |  |
| < 0.37 | 28 | 100 |  | Reference |  |
| 0.37-0.80 | 29 | 93 |  | 1.11 (0.54-2.28) | 0.769 |
| ≥0.80 | 39 | 59 |  | 2.34 (1.15-4.75) | 0.019 |
| *P* value for trend |  |  |  |  | 0.019 |
|  |  |  |  |  |  |
| IFN-γ, pg/mL |  |  |  |  |  |
| <21.86 | 30 | 111 |  | Reference |  |
| 21.86-49.57 | 25 | 87 |  | 1.00 (0.49-2.04) | 0.996 |
| ≥49.57 | 42 | 55 |  | 2.32 (1.16-4.64) | 0.017 |
| *P* value for trend |  |  |  |  | 0.019 |
|  |  |  |  |  |  |
| IL-10, pg/mL |  |  |  |  |  |
| < 2.24 | 29 | 111 |  | Reference |  |
| 2.24-8.95 | 28 | 84 |  | 1.27 (0.62-2.57) | 0.512 |
| ≥8.95 | 38 | 59 |  | 2.09 (1.04-4.20) | 0.039 |
| *P* value for trend |  |  |  |  | 0.041 |
|  |  |  |  |  |  |
| IL-9 , pg/mL |  |  |  |  |  |
| <12.41 | 27 | 109 |  | Reference |  |
| 12.41-37.27 | 28 | 91 |  | 1.38 (0.69-2.77) | 0.367 |
| ≥37.27 | 42 | 53 |  | 2.38 (1.18-4.77) | 0.015 |
| *P* value for trend |  |  |  |  | 0.016 |
|  |  |  |  |  |  |
| IL-6, pg/mL |  |  |  |  |  |
| < 9.40 | 26 | 117 |  | Reference |  |
| 9.40-30.10 | 31 | 84 |  | 1.60 (0.79-3.20) | 0.190 |
| ≥30.10 | 40 | 52 |  | 2.70 (1.33-5.48) | 0.006 |
| *P* value for trend |  |  |  |  | 0.006 |
|  |  |  |  |  |  |
| IL-4, pg/mL |  |  |  |  |  |
| < 0.16 | 33 | 100 |  | Reference |  |
| 0.16-0.39 | 25 | 91 |  | 0.81 (0.40-1.62) | 0.547 |
| ≥0.39 | 39 | 63 |  | 1.55 (0.78-3.08) | 0.208 |
| *P* value for trend |  |  |  |  | 0.234 |

AF, atrial fibrillation; OR, odds ratio; CI, confidence interval; IL-17A, interleukin 17A; IL-17F, interleukin 17F; IL-21, interleukin 21; IL-22, interleukin 22; IFN-γ, interferon-γ; IL-10, interleukin 10; IL-9, interleukin 9; IL-6, interleukin 6; IL-4, interleukin 4.

* The cytokine levels were analyzed as ordinal categorical variables using tertiles.

† A multivariable unconditional logistic regression analysis was used to estimate the OR (95% CI) adjusted for age, gender, history of hypertension, history of diabetes, smoking status, drinking status, body mass index (BMI), history of coronary heart disease, use of statins and aspirin.

Supplementary Table S4. Associations between Th17-related cytokine levels with AF in participants aged at least 60 years

| Cytokines ^*^ | Sample sizes, n | |  | Unconditional logistic regression† | |
| --- | --- | --- | --- | --- | --- |
|  | AF | Control |  | OR (95% CI) | *P* value |
| IL-17A , pg/mL |  |  |  |  |  |
| < 16.30 | 59 | 32 |  | Reference |  |
| 16.30-34.27 | 74 | 26 |  | 1.72 (0.88-3.35) | 0.110 |
| ≥34.27 | 106 | 24 |  | 2.38 (1.24-4.56) | 0.009 |
| *P* value for trend |  |  |  |  | 0.010 |
|  |  |  |  |  |  |
| IL-17F, pg/mL |  |  |  |  |  |
| < 0.01 | 135 | 61 |  | Reference |  |
| 0.01-0.02 | 49 | 7 |  | 3.03 (1.25-7.34) | 0.014 |
| ≥0.02 | 54 | 14 |  | 1.61 (0.81-3.22) | 0.175 |
| *P* value for trend |  |  |  |  | 0.064 |
|  |  |  |  |  |  |
| IL-21, pg/mL |  |  |  |  |  |
| < 27.62 | 67 | 32 |  | Reference |  |
| 27.62-50.66 | 78 | 31 |  | 1.26 (0.67-2.37) | 0.477 |
| ≥50.66 | 93 | 19 |  | 2.18 (1.11-4.30) | 0.025 |
| *P* value for trend |  |  |  |  | 0.025 |
|  |  |  |  |  |  |
| IL-22 , pg/mL |  |  |  |  |  |
| < 0.37 | 65 | 35 |  | Reference |  |
| 0.37-0.80 | 78 | 25 |  | 1.70 (0.88-3.29) | 0.115 |
| ≥0.80 | 95 | 22 |  | 2.20 (1.14-4.24) | 0.018 |
| *P* value for trend |  |  |  |  | 0.018 |
|  |  |  |  |  |  |
| IFN-γ, pg/mL |  |  |  |  |  |
| <21.86 | 58 | 29 |  | Reference |  |
| 21.86-49.57 | 78 | 30 |  | 1.55 (0.80-3.01) | 0.192 |
| ≥49.57 | 102 | 23 |  | 2.13 (1.09-4.15) | 0.027 |
| *P* value for trend |  |  |  |  | 0.028 |
|  |  |  |  |  |  |
| IL-10, pg/mL |  |  |  |  |  |
| < 2.24 | 58 | 28 |  | Reference |  |
| 2.24-8.95 | 78 | 28 |  | 1.54 (0.78-3.05) | 0.210 |
| ≥8.95 | 101 | 22 |  | 2.24 (1.14-4.42) | 0.020 |
| *P* value for trend |  |  |  |  | 0.020 |
|  |  |  |  |  |  |
| IL-9 , pg/mL |  |  |  |  |  |
| <12.41 | 62 | 28 |  | Reference |  |
| 12.41-37.27 | 69 | 35 |  | 0.99 (0.52-1.89) | 0.968 |
| ≥37.27 | 106 | 18 |  | 2.48 (1.24-4.99) | 0.011 |
| *P* value for trend |  |  |  |  | 0.010 |
|  |  |  |  |  |  |
| IL-6, pg/mL |  |  |  |  |  |
| < 9.40 | 57 | 28 |  | Reference |  |
| 9.40-30.10 | 75 | 30 |  | 1.29 (0.66-2.53) | 0.451 |
| ≥30.10 | 106 | 24 |  | 2.04 (1.05-3.97) | 0.035 |
| *P* value for trend |  |  |  |  | 0.033 |
|  |  |  |  |  |  |
| IL-4, pg/mL |  |  |  |  |  |
| < 0.16 | 73 | 34 |  | Reference |  |
| 0.16-0.39 | 71 | 25 |  | 1.43 (0.74-2.75) | 0.288 |
| ≥0.39 | 95 | 23 |  | 1.85 (0.97-3.50) | 0.060 |
| *P* value for trend |  |  |  |  | 0.060 |

AF, atrial fibrillation; OR, odds ratio; CI, confidence interval; IL-17A, interleukin 17A; IL-17F, interleukin 17F; IL-21, interleukin 21; IL-22, interleukin 22; IFN-γ, interferon-γ; IL-10, interleukin 10; IL-9, interleukin 9; IL-6, interleukin 6; IL-4, interleukin 4.

* The cytokine levels were analyzed as ordinal categorical variables using tertiles.

† A multivariable unconditional logistic regression analysis was used to estimate the OR (95%CI) adjusted for age, gender, history of hypertension, history of diabetes, smoking status, drinking status, body mass index (BMI), history of coronary heart disease, use of statins and aspirin.

Supplementary Table S5. Characteristics of all patients with AF and controls aged at least 51 years

| Characteristics * | AF cases  (n=336) | Controls  (n=126) | P value † |
| --- | --- | --- | --- |
| Age, y | 67 (58-74) | 64 (56-73) | 0.179 |
| Gender, n (%) |  |  | 0.662 |
| Male | 181 (53.9) | 65 (51.6) |  |
| Female | 155 (46.1) | 61 (48.4) |  |
| Hypertension, n (%) |  |  | < 0.001 |
| Yes | 163 (48.5) | 85 (67.5) |  |
| No | 173 (51.5) | 41 (32.5) |  |
| Diabetes, n (%) |  |  | 0.445 |
| Yes | 49 (14.6) | 22 (17.5) |  |
| No | 287 (85.4) | 104 (82.5) |  |
| Smoking status, n (%) |  |  | 0.592 |
| Smoker | 121 (36.0) | 42 (33.3) |  |
| Non-smoker | 215 (64.0) | 84 (66.7) |  |
| Drinking status, n (%) |  |  | 0.063 |
| Drinker | 110 (32.7) | 30 (23.8) |  |
| Non-drinker | 226 (67.3) | 96 (76.2) |  |
| BMI status, n (%) |  |  | 0.766 |
| BMI<18.5 | 15 (4.5) | 3 (2.4) |  |
| 18.5≤BMI<25.0 | 190 (56.5) | 74 (58.7) |  |
| 25.0≤BMI<30.0 | 105 (31.3) | 40 (31.7) |  |
| BMI≥30.0 | 26 (7.7) | 9 (7.1) |  |
| Taking statins, n (%) |  |  | 0.193 |
| Yes | 41 (12.2) | 10 (7.9) |  |
| No | 295 (87.8) | 116 (92.1) |  |
| Taking aspirin, n (%) |  |  | 0.124 |
| Yes | 34 (10.1) | 7 (5.6) |  |
| No | 302 (89.9) | 119 (94.4) |  |
| Coronary artery disease |  |  | 0.837 |
| Yes | 75 (22.3) | 27 (21.4) |  |
| No | 261 (77.7) | 99 (78.6) |  |
| IL-17A, pg/mL | 31.00 (15.98-46.73) | 20.34 (13.98-34.49) | <0.001 |
| IL-17F, pg/mL | 0.01 (0.01-0.02) | 0.01 (0.01-0.02) | 0.004 |
| IL-21, pg/mL | 44.48 (25.33-67.13) | 35.05 (18.59-51.76) | 0.002 |
| IL-22, pg/mL | 0.69 (0.32-0.95) | 0.48 (0.10-0.83) | 0.002 |
| IFN-γ, pg/mL | 42.59 (21.86-67.13) | 29.17 (14.84-49.84) | <0.001 |
| IL-10, pg/mL | 7.53 (2.08-15.73) | 3.26 (0.90-9.76) | <0.001 |
| IL-9, pg/mL | 33.08 (11.89-56.25) | 16.05 (7.90-33.44) | <0.001 |
| IL-6, pg/mL | 26.45 (9.98-47.47) | 12.70 (3.70-32.90) | <0.001 |
| IL-4, pg/mL | 0.33 (0.11-0.54) | 0.21(0.08-0.40) | <0.001 |

AF, atrial fibrillation; IQR, interquartile range; BMI, body mass index; IL-17A, interleukin 17A; IL-17F, interleukin 17F; IL-21, interleukin 21; IL-22, interleukin 22; IFN-γ, interferon-γ; IL-10, interleukin 10; IL-9, interleukin 9; IL-6, interleukin 6; IL-4, interleukin 4.

*Entries are n (%) for categorical variables and median (5th percentile-75th percentile) for continuous variables as appropriate.

† Chi-square test for categorical variables, and Mann-Whitney U test for continuous variables.

Supplementary Table S6. Associations between Th17-related cytokine levels with AF in patients with AF and controls aged at least 51 years

| Cytokines ^*^ | Sample sizes, n | |  | Unconditional logistic regression† | |
| --- | --- | --- | --- | --- | --- |
|  | AF | Control |  | OR (95% CI) | *P* value |
| IL-17A , pg/mL |  |  |  |  |  |
| < 17.75 | 96 | 59 |  | Reference |  |
| 17.75-38.76 | 114 | 43 |  | 1.76 (1.07-2.90) | 0.027 |
| ≥38.76 | 126 | 24 |  | 3.10 (1.77-5.45) | <0.001 |
| *P* value for trend |  |  |  |  | <0.001 |
|  |  |  |  |  |  |
| IL-17F, pg/mL |  |  |  |  |  |
| < 0.01 | 184 | 91 |  | Reference |  |
| 0.01-0.02 | 68 | 12 |  | 2.77 (1.40-5.50) | 0.004 |
| ≥0.02 | 82 | 23 |  | 1.79 (1.04-3.10) | 0.037 |
| *P* value for trend |  |  |  |  | 0.010 |
|  |  |  |  |  |  |
| IL-21, pg/mL |  |  |  |  |  |
| < 28.84 | 105 | 50 |  | Reference |  |
| 28.84-54.06 | 104 | 50 |  | 1.06 (0.64-1.74) | 0.821 |
| ≥54.06 | 126 | 26 |  | 2.24 (1.28-3.91) | 0.005 |
| *P* value for trend |  |  |  |  | 0.006 |
|  |  |  |  |  |  |
| IL-22 , pg/mL |  |  |  |  |  |
| < 0.40 | 99 | 57 |  | Reference |  |
| 0.40-0.83 | 117 | 38 |  | 1.83 (1.10-3.07) | 0.021 |
| ≥0.83 | 118 | 31 |  | 2.27 (1.33-3.88) | 0.003 |
| *P* value for trend |  |  |  |  | 0.019 |
|  |  |  |  |  |  |
| IFN-γ, pg/mL |  |  |  |  |  |
| <25.43 | 97 | 57 |  | Reference |  |
| 25.43-56.45 | 113 | 45 |  | 1.67 (1.01-2.76) | 0.044 |
| ≥56.45 | 125 | 24 |  | 2.98 (1.69-5.25) | <0.001 |
| *P* value for trend |  |  |  |  | <0.001 |
|  |  |  |  |  |  |
| IL-10, pg/mL |  |  |  |  |  |
| < 2.73 | 98 | 55 |  | Reference |  |
| 2.73-10.65 | 109 | 43 |  | 1.42 (0.85-2.37) | 0.184 |
| ≥10.65 | 125 | 24 |  | 2.82 (1.59-4.98) | <0.001 |
| *P* value for trend |  |  |  |  | <0.001 |
|  |  |  |  |  |  |
| IL-9 , pg/mL |  |  |  |  |  |
| <14.23 | 100 | 56 |  | Reference |  |
| 14.23-42.45 | 110 | 46 |  | 1.44 (0.88-2.37) | 0.145 |
| ≥42.45 | 124 | 23 |  | 2.84 (1.60-5.02) | <0.001 |
| *P* value for trend |  |  |  |  | <0.001 |
|  |  |  |  |  |  |
| IL-6, pg/mL |  |  |  |  |  |
| < 11.10 | 96 | 59 |  | Reference |  |
| 11.10-35.10 | 112 | 42 |  | 1.72 (1.04-2.86) | 0.035 |
| ≥35.10 | 127 | 25 |  | 3.07 (1.76-5.35) | <0.001 |
| *P* value for trend |  |  |  |  | <0.001 |
|  |  |  |  |  |  |
| IL-4, pg/mL |  |  |  |  |  |
| < 0.14 | 106 | 52 |  | Reference |  |
| 0.14-0.41 | 106 | 45 |  | 1.18 (0.72-1.95) | 0.514 |
| ≥0.41 | 124 | 29 |  | 2.01 (1.17-3.45) | 0.012 |
| *P* value for trend |  |  |  |  | 0.013 |

AF, atrial fibrillation; OR, odds ratio; CI, confidence interval; IL-17A, interleukin 17A; IL-17F, interleukin 17F; IL-21, interleukin 21; IL-22, interleukin 22; IFN-γ, interferon-γ; IL-10, interleukin 10; IL-9, interleukin 9; IL-6, interleukin 6; IL-4, interleukin 4.

* The analysis was conducted on all patients with AF and controls aged at least 51 years.

The cytokine levels were analyzed as ordinal categorical variables using tertiles.

† A multivariable unconditional logistic regression analysis was used to estimate the OR (95% CI) adjusted for age, gender, history of hypertension, history of diabetes, smoking status, drinking status, body mass index (BMI), history of coronary heart disease, use of statins and aspirin.
